# Supplementary material for: Hybrid Models and Biological Model Reduction with PyDSTool
Source: PLoS Comput Biol. 2012 Aug 9;8(8):e1002628. doi: 10.1371/journal.pcbi.1002628 (PMC3415397; doi:10.1371/journal.pcbi.1002628)
Supplement: Text S4 — Complete source code for the PyDSTool package (version 0.88.120504). Includes API documentation and help files linking to web pages. This file is identical to the current public release on Sourceforge.net. (ZIP) [file pcbi.1002628.s004.zip › PyDSTool/html/toc-PyDSTool.Toolbox.DSSRT_tools-module.html]

xml version="1.0" encoding="ascii"?


DSSRT\_tools


# Module DSSRT\_tools

---

## Classes

DSSRT\_info  

## Functions

plotNetworkGraph  

## Variables

ALLOW\_THREADS

Abs

Acos

Asin

Atan

Atan2

BUFSIZE

Betavariate

CFGorder

CLIP

Ceil

Choice

Cos

Cosh

Degrees

E

ERR\_CALL

ERR\_DEFAULT

ERR\_DEFAULT2

ERR\_IGNORE

ERR\_LOG

ERR\_PRINT

ERR\_RAISE

ERR\_WARN

Exp

Expovariate

FLOATING\_POINT\_SUPPORT

FPE\_DIVIDEBYZERO

FPE\_INVALID

FPE\_OVERFLOW

FPE\_UNDERFLOW

Fabs

False\_

Floor

Fmod

Frexp

Gammavariate

Gauss

Getrandbits

Getstate

Hypot

Infinity

Jumpahead

Ldexp

Log

Log10

Lognormvariate

MAXDIMS

Max

Min

Modf

NAN

NINF

NZERO

Normalvariate

PINF

PZERO

Paretovariate

Pi

Pow

RAISE

Radians

Randint

Random

Randrange

SHIFT\_DIVIDEBYZERO

SHIFT\_INVALID

SHIFT\_OVERFLOW

SHIFT\_UNDERFLOW

Sample

ScalarType

Seed

Setstate

Shuffle

Sin

Sinh

Sqrt

Sum

Systemrandom

Tan

Tanh

True\_

UFUNC\_BUFSIZE\_DEFAULT

UFUNC\_PYVALS\_NAME

Uniform

Vonmisesvariate

WRAP

Weibullvariate

Wichmannhill

absolute

add

bitwise\_and

bitwise\_not

bitwise\_or

bitwise\_xor

c\_

cast

conj

conjugate

copysign

deg2rad

divide

equal

exp2

expm1

floor\_divide

fmax

fmin

greater\_equal

index\_exp

inf

infty

invert

isinf

left\_shift

little\_endian

log1p

logaddexp

logaddexp2

logical\_and

logical\_not

logical\_xor

maximum

mgrid

minimum

multiply

n

nan

nbytes

negative

newaxis

nextafter

nodes\_order

not\_equal

ogrid

ones\_like

r\_

rad2deg

reciprocal

remainder

right\_shift

rint

s\_

sctypeDict

sctypeNA

sctypes

signbit

spacing

square

subtract

t

true\_divide

trunc

typeDict

typeNA

typecodes

---

[hide private]
